# Repetition Suppression for Noisy and Intact Faces in the Occipito-Temporal Cortex

**DOI:** 10.3389/fpsyg.2019.01348

**Published:** 2019-06-14

**Authors:** Sophie-Marie Rostalski, Catarina Amado, Gyula Kovács

**Affiliations:** ^1^Department of Biological Psychology and Cognitive Neurosciences, Institute of Psychology, Friedrich Schiller University Jena, Jena, Germany; ^2^Department of Computer Science, Experimental Cognitive Science Research Group, Eberhard Karls Universität Tübingen, Tübingen, Germany

**Keywords:** repetition suppression, predictive coding, precision, noise, fusiform face area

## Abstract

Repetition suppression (RS), the relative lower neural response magnitude to repeated as compared to non-repeated stimuli, is often explained within the predictive coding framework. According to this theory, precise predictions (priors) together with less precise sensory evidences lead to decisions that are determined largely by the predictions and the other way around. In other words, the prediction error, namely the magnitude of RS, should depend on the precision of predictions and sensory inputs. In the current study, we aimed at testing this idea by manipulating the clarity and thereby the precision of the incoming sensory data by adding noise to the images. This resulted in an fMRI adaptation design with repeated or alternating trials showing clear or noisy face stimuli. Our results show a noise effect on the activity in the fusiform face area (FFA), namely less activation for noisy than for clear trials, which supports previous findings. No such effects could be found in OFA or LO. Data also showed reliable RS in the FFA (bilateral) and unilaterally in OFA (right) and LO (left). Interestingly, the noise added to the stimuli did not affect the magnitude of RS in any of the tested cortical areas. This suggests that the clarity of the sensory input is not crucial in determining the magnitude of RS.

## Introduction

Repetition suppression (RS), the relative lower neural response magnitude to repeated as compared to non-repeated stimuli, is one of the most studied phenomena of cognitive neurosciences. Over the last years, not only RS but other stimulus repetition-related phenomena, such as expectation suppression or surprise-related response elevation, were explained under the framework of predictive coding (Summerfield et al., 2008; Todorovic and de Lange, 2012; Grotheer and Kovacs, 2014; Mayrhauser et al., 2014; Grotheer and Kovács, 2015). This theory states that perception is not determined solely by the incoming stimuli themselves, but it is also modulated by inferential processes (Rao and Ballard, 1999). In other words, the sensory inputs together with our prior experiences are used to form predictions of upcoming events to ensure efficient processing (Friston, 2005). For a better understanding of these processes, several prior studies manipulated the temporal context of a stimulus to alter predictions (Auksztulewicz and Friston, 2016; Grotheer and Kovács, 2016). These studies suggested that stimulus repetitions lead to lower prediction errors and this is manifest in RS while rarely presented, thereby surprising, stimuli lead to higher prediction errors and enhanced neural responses (for a review, see Grotheer and Kovács, 2016). Although recently, numerous studies explained repetition and expectation-related phenomena under the framework of predictive coding, this explanation is not unchallenged in the literature. While expectations seem capable of modulating RS in many cases, RS and expectation suppression (ES) were dissociated from each other in several studies (Todorovic and de Lange, 2012; Grotheer and Kovács, 2015; Feuerriegel et al., 2018) and therefore seem to reflect different neuronal mechanisms. Further, human fMRI studies with objects (Kovacs et al., 2013; Grotheer and Kovacs, 2014) and nonhuman primate single-cell studies with objects as well as recent single-cell (Vinken et al., 2018) or fMRI (Olkkonen et al., 2017) studies with faces failed to find any trace of modulatory effects of expectation on RS (see, however, Mayrhauser et al., 2014 and Kronbichler et al., 2018 for a different conclusion). Therefore, the role of top-down modulatory effects, such as predictions and expectation, in determining the magnitude of RS is under heavy discussion as of today.

Although RS seems to be a robust phenomenon, that has been investigated in several paradigms (for reviews, see Grill-Spector et al., 2006; Krekelberg et al., 2006), there are evidences for repetition enhancement (i.e., an enhanced neural response for repeated stimuli) as well (Henson, 2003; Turk-Browne et al., 2007; De Gardelle et al., 2013; Segaert et al., 2013; Recasens et al., 2015). For example, Turk-Browne et al. (2007) could show that the attenuating effect on the BOLD responses of showing two identical scenes compared to two different scenes in one trial could be reversed by reducing the contrast of the stimuli. This modulatory effect on neural responses to repetitions is introducing an important factor into the RS research field, namely precision or clarity of visual input.

Prior studies (Auksztulewicz and Friston, 2016) conceptualized prediction error as the magnitude of neural responses in certain “error units.” The repeated presentations of a given stimulus would, in turn, reduce the activity of these neurons, leading to RS. According to theories of predictive coding, precise predictions (priors) together with less precise sensory evidences lead to decisions that are determined largely by the predictions; in other words, the prediction error is increased if predictions fail to come true. This would in turn reduce the magnitude of RS for noisy when compared to clear sensory inputs. However, if the predicted priors are less precise (for example due to the frequent occurrence of unexpected events) but the incoming sensory stimuli are clear and precise, then the *a posteriori* decisions are rather determined by the sensory stimulation and the prediction errors are lower (O’Reilly et al., 2012; Adams et al., 2013; Auksztulewicz and Friston, 2016; Sterzer et al., 2018).

**Figure 1:**
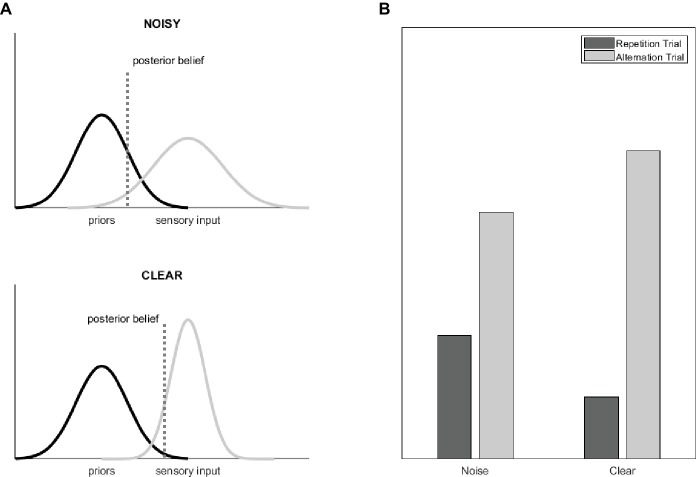
**(A)** The graphs show the Gaussian probability distributions that represent the distribution of the priors (i.e., the *a priori* beliefs, black) and of the sensory evidences (gray) as well as the resulting posterior beliefs (dotted line) for a situation where the statistics-based priors are kept constant and the precision of the sensory stimuli is modulated (for example by adding noise to the images). Precision can be understood as the inverse of the distribution width. Reducing the relative precision of the sensory input biases the posterior beliefs toward the priors and thereby reduces predictive error (figure adapted from Adams et al., 2013). **(B)** Theoretical BOLD signal magnitudes for alternating and repeating stimulus pairs and for stimuli with and without noise, separately. Note that these results assume that predictive error is reflected in the magnitude of RS (Grotheer and Kovács, 2016).

In the current study, we aimed at testing this idea by manipulating sthe precision of the incoming sensory data by adding noise to the images. We reasoned that the modulatory effect of stimulus precision on prediction errors might be reflected in the magnitude of RS. Because sensory uncertainty is assumed to reduce the difference of priors and posterior beliefs (Figure 1A; Adams et al., 2013; Sterzer et al., 2018), a smaller prediction error (RS magnitude) is expected for noisy, as compared to clear visual stimuli. In other words, alternations lead to much higher prediction errors and repetitions to lower prediction errors when the sensory input is clear as compared to noisy, which results in higher RS magnitude (Figure 1B) for clear visual inputs.

Indeed, previous studies suggest the differential processing of noisy stimuli (Wild and Busey, 2004; Banko et al., 2011). For example, Banko et al. (2011) manipulated task difficulty by decreasing the phase coherence of face stimuli and found that this affects early electrophysiological responses. The visually evoked P1 showed a higher amplitude to noisy stimuli, whereas the face-sensitive N170 showed a lower amplitude. In line with the P1 modulation, fMRI data showed increased activation in the lateral occipital cortex (LO) due to noise (Banko et al., 2011). Also, Hermann et al. (2015) found that noisy stimuli with lower phase coherence lead to increased activity in the LO. This suggests increased processing demands in the visual cortex due to added visual noise. However, authors also found reduced activity in the face-selective fusiform face area (FFA) when noise was added (Hermann et al., 2015). In addition, a linear increase in the amplitude of a face-sensitive ERP component (N170) (Jemel et al., 2003) could be observed by decreasing the level of a Gaussian distributed noise, added to face stimuli gradually. This result could later be confirmed with fMRI data by Horner and Andrews (2009) who manipulated phase coherence and found evidence for the principle of scaling for preferred stimuli in the FFA. This suggests the linearity of the BOLD response and the noise level in face stimuli. Altogether, these studies show that visual noise indeed affects neural processing, but it is not clear what impact that effect has on inferential processes and subsequent predictions.

To the best of our knowledge so far, no study compared the effect of stimulus repetitions for noisy and clear stimuli in the ventral temporal cortex. Therefore, in the present study, noise was added to face stimuli to manipulate the precision of sensory stimulation in a design containing repeated and alternating trials. Trials could therefore either consist of pairs of clear or noisy faces, which could either be the same or different. Activity in face-specific areas (FFA and OFA, occipital face area) as well as in LO was acquired using fMRI. Based on prior evidences (Horner and Andrews, 2009; Hermann et al., 2015), noisy stimuli were expected to elicit lower BOLD responses than clear ones in the regions of fusiform gyrus, but an enhanced response was expected in the lateral occipital regions (Banko et al., 2011; Hermann et al., 2015). Also, in line with the predictive coding theory, repeated trials were hypothesized to show a smaller neuronal response than alternating trials. We reasoned that this RS effect should be modulated by the clarity of the stimuli if predictions are indeed less precise for noisy as compared to clear stimuli (Auksztulewicz and Friston, 2016).

## Materials and Methods

### Participants

Twenty-three subjects participated in this study. One subject was excluded from the analysis due to excessive movements during image acquisition. The remaining 22 participants (11 females, one left-handed and one both left- and right-handed) were between 18 and 31 years of age (*M* = 22; SD = 3.81) and all had normal or corrected to normal vision. Previous fMRI studies, using stimulus pairs and reporting significant RS, as well as significant predictive modulations of RS (Summerfield et al., 2008; Kovacs et al., 2013; Grotheer et al., 2014; Grotheer and Kovacs, 2014) were typically able to find modulatory effects of RS by other factors, such as probabilistic predictions with sample sizes between 11 and 26. Therefore, here, we reasoned, that with the tested number of participants, we could reliably detect any interaction of noise and RS and this is supported by the results of the Bayes factor analyses.

Participants were fully informed about the study and gave written consents to participate. They received course credits for participation. The experiment was conducted in accordance with the guidelines of the Declaration of Helsinki, and with the approval of the ethics committee of the University of Jena.

### Stimuli and Procedure

A total set of 490 unfamiliar faces (246 clear (127 female) and 244 noisy (127 female)) were used as stimuli. Those were shown in the center of the screen with a superimposed grey scale mask, which additionally covered the hair and the shape of the face resulting in round-shaped faces including eyes, nose, and mouth. Noisy stimuli were generated by superimposing Fourier-transformed versions of the original images on the faces where phase coherence was reduced (45%) by the weighted mean phase technique (Dakin et al., 2002).

**Figure 2:**
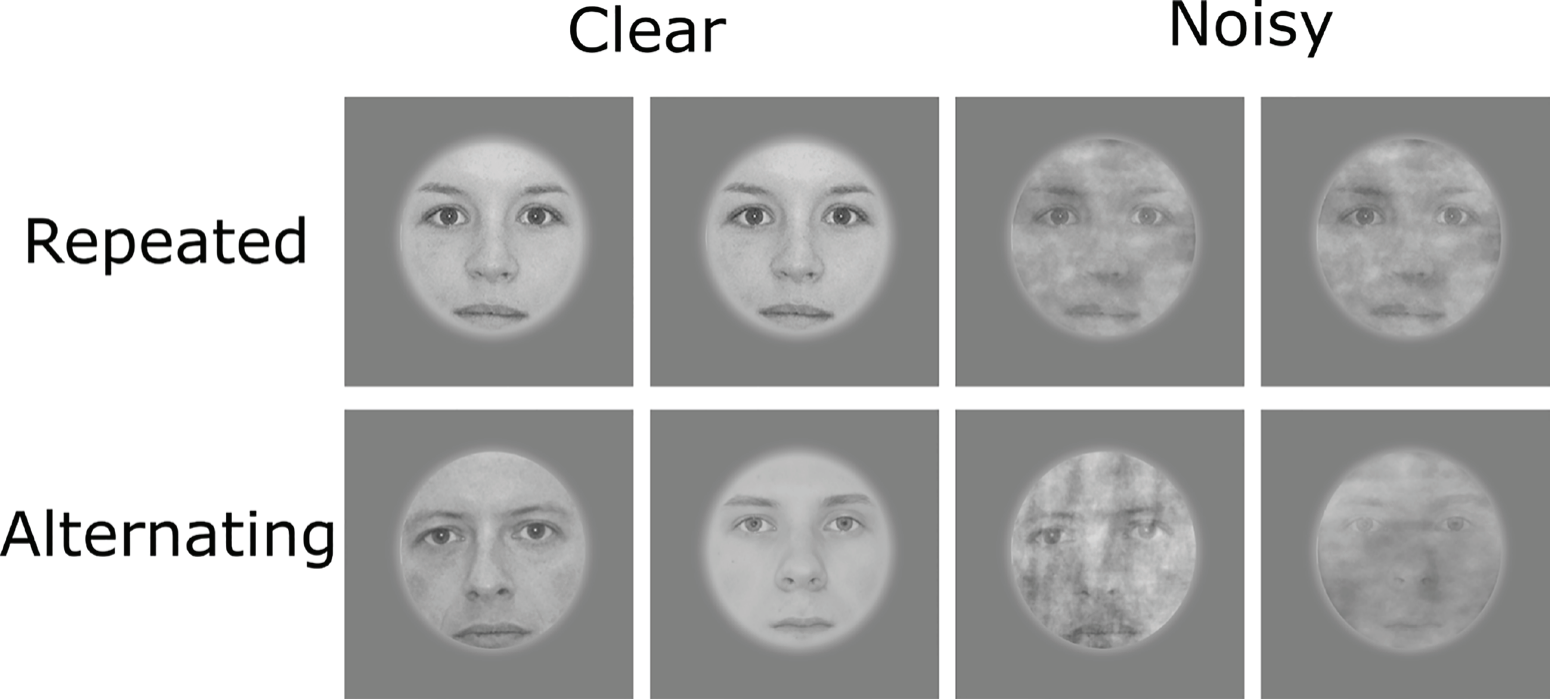
Examples for the four possible trial types (excluding target trials). Written informed consent for publishing these images was given by the respective persons.

**Figure 3:**
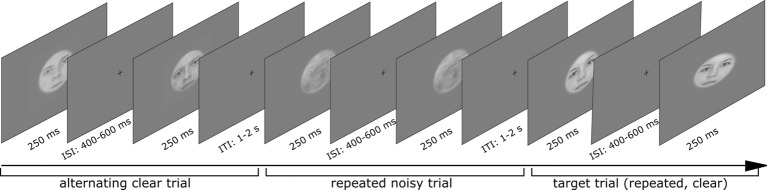
Sequence of three trials including clear alternating, noisy repeated, and one target trial (clear repeated). Written informed consent for publishing these images was given by the respective persons.

Participants completed three experimental runs, each including 120 trials of the four different trial types (Figure 2) in a randomized fashion. One trial included two stimulus presentations, which could either be the same face (repeated trial) or two different faces (alternating trial). Participants’ task was to detect target trials, in which the second face stimulus was tilted by 10° either clockwise or counterclockwise and to indicate this direction by pressing a button (Figure 3). Such target trials were equally distributed across the four conditions and represented 20% of the overall trial amount and were excluded from any further analysis.

For defining regions of interests (ROI), a localizer sequence was performed. Grayscale images of faces, objects, and Fourier-transformed noise patterns were presented (exposition time: 300 ms, interstimulus interval: 200 ms) in blocks of 20 s intermitted by a break of 20 s and were repeated four times.

### Imaging Parameters

Neuroimaging was performed using a Prisma fit 3 T MRI Scanner from Siemens. During the functional runs, T2*-weighted images (35 slices, TR = 2,000 ms, TE = 30 ms, isotropic voxel size of 3 mm) were acquired continuously. High-resolution T1*-weighted simages (TR = 2,300 ms, TE = 3.03 ms, isotropic voxel size of 1 mm) were acquired to obtain a 3D structural scan. Data were preprocessed using SPM12 (Wellcome Trust Centre for Neuroimaging, University College London, UK). The functional images were slice-timed, realigned, co-registered to the structural scan, and afterward normalized to the MNI space and smoothed using an 8-mm Gaussian kernel.

ROIs were defined using the data from the localizer sequence and canonical hemodynamic response functions (HRFs) were extracted using MarsBaR (Brett et al., 2002). HRFs were estimated for all subjects and ROIs. Then, peak values were submitted to repeated measurement ANOVAs with the factors noise level (clear vs. noisy) and repetition (repeated vs. alternating).

## Results

### Behavioral Results

A repeated measurements ANOVA with the factors noise (*clear* vs. *noisy*) and trial type (*repeated* vs. *alternating*) was conducted for the reaction times and accuracy. Regarding the reaction times, no significant effect was revealed from the analysis. However, a significant main effect for noise level was found for the accuracy rates, *F*(1,21) = 12.00, *p* < 0.01, *η* = 0.36, which shows better performance for clear (*M* = 94.4%, SD = 10.3%) as compared to noisy trials (*M* = 88.9%, s = 12.3%).

### Neuroimaging Results

**Figure 4:**
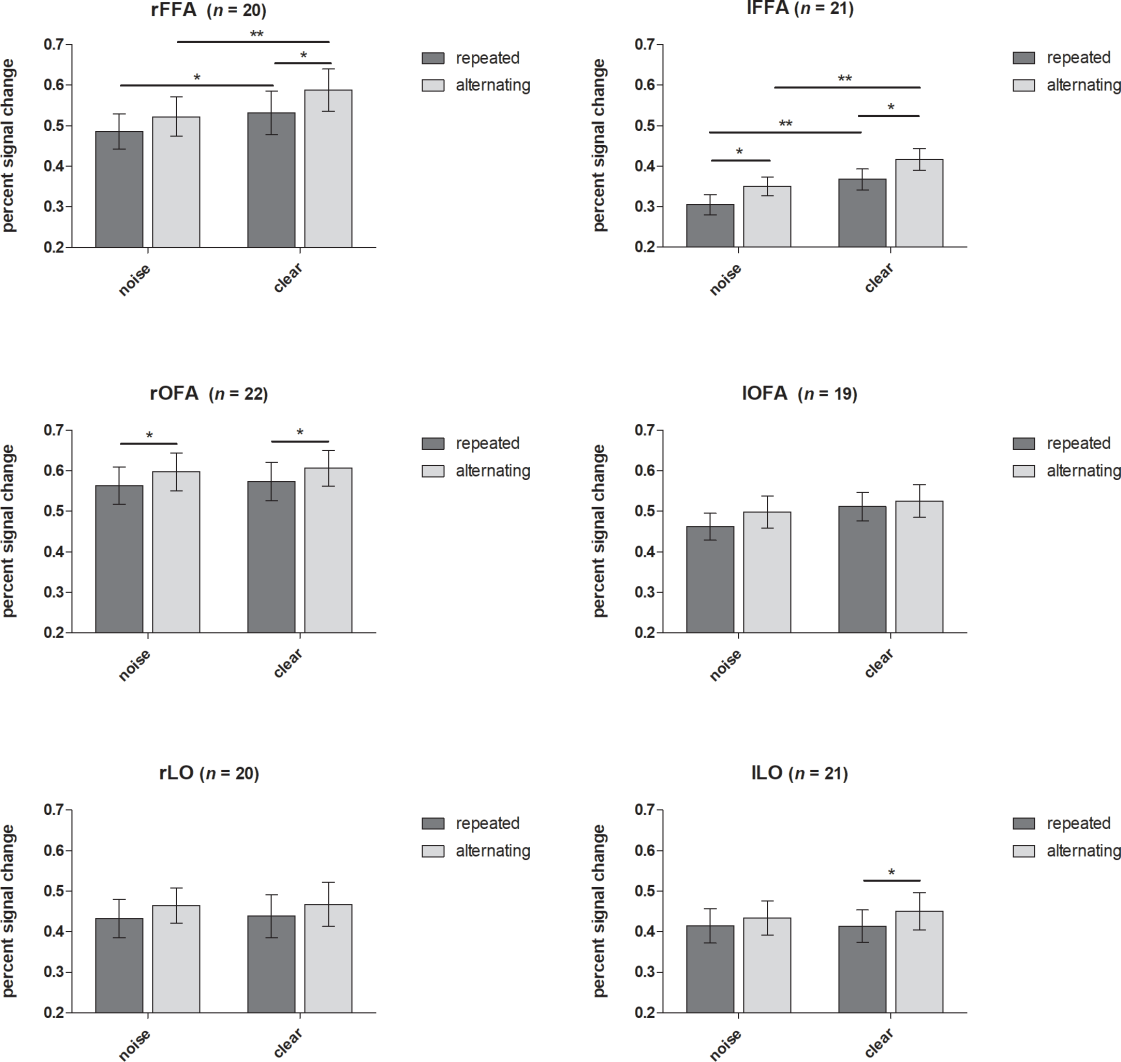
Effects of noise and repetitions. Percent signal changes of FFA, OFA, and LO (left and right hemispheres for each) are presented for condition and trial type. Error bars indicate standard errors. Displayed significant differences refer to Fisher’s LSD *post hoc* test. *p < 0.05, **p < 0.01.

Neuroimaging results are depicted in Figure 4. We found a similar pattern in the FFA of the two hemispheres. A significant main effect of noise level was found, *F*(1,19) = 14.18, *p* < 0.01, *η* = 0.43 for right hemisphere and *F*(1,20) = 19.45, *p* < 0.001, *η* = 0.49 for left hemisphere, with clear trials eliciting larger BOLD signal than the noisy ones. Additionally, a significant main effect of trial type was observed in both hemispheres: *F*(1,19) = 16.22, *p* < 0.001, *η* = 0.46 for right hemisphere and *F*(1,20) = 15.99, *p* < 0.001, *η* = 0.44 for left hemisphere. This effect suggests a generally higher signal for alternating as compared to repeated trials. However, no interaction between noise level and trial type was found, neither for the right, *F*(1,19) < 1, *p* = 0.44, *η* = 0.03, nor for the left hemisphere, *F*(1,20) < 1, *p* = 0.87, *η* = 0.00, suggesting that the observed RS is similar for noisy and clear stimuli in the FFA.

The same analysis performed on the right OFA revealed a significant main effect of trial type for the right hemisphere, *F*(1,21) = 8.14, *p* < 0.01, *η* = 0.28, showing that alternating trials elicit greater signal changes than repeated ones. However, the main effect of noise remained nonsignificant, *F*(1,21) < 1, *p* = 0.54, *η* = 0.02, as well as the interaction effect did, *F*(1,21) < 1, *p* = 0.94, *η* = 0.00. The same analysis for the left OFA revealed no significant main effect or interaction.

In the LO, similar to the OFA, the main effect of trial type was found to be significant in one hemisphere, the left one only, *F*(1,20) = 4.60, *p* < 0.05, *η* = 0.19. Again, repetitions led to lower signal than alternations in the LO as well whereas the right hemisphere showed a strong tendency in this direction only, *F*(1,20) = 3.59, *p* = 0.07, *η* = 0.16. No other main effect or interaction reached significance.

We additionally evaluated the likelihood that there is no interaction between the two factors using a Bayesian repeated measures ANOVA to substantiate our conclusion. This analysis, performed in JASP (JASP Team, 2018), provides the Bayes factor, reflecting how much more likely a dataset reflects the null hypothesis compared to the alternative hypotheses. To get the Bayes factor for the interaction, we performed the division of BF01 of the model containing the two main effects and the interaction between these by the model containing the two main effects only. Values reported here show the Bayes factor for the null hypothesis against the hypothesis of an interaction.

The estimated Bayes factor (null/alternative) for an interaction of condition and trial type in the right FFA was 2.6, suggesting that the null hypothesis of no interaction is 2.6 times more likely than the alternative hypothesis. Bayes factor (null/alternative) for an interaction effect in the left FFA was 3.1, providing substantial evidence for the null hypothesis.

Regarding the right OFA, a Bayes factor of 3.3, meaning that data are 3.3 times more likely to occur under the null hypothesis, provides substantial evidence against the presence of an interaction effect between the two factors. In the left OFA, the calculated Bayes factor for the interaction between condition and trial type was 2.9, implying that the null hypothesis of no interaction is 2.9 times more likely than the alternative hypothesis.

The Bayesian repeated measures ANOVA in the right LO revealed a Bayes factor of 3.1, providing substantial evidence for the null hypothesis, that there is no interaction between the two factors noise level and trial type. For the left LO, the Bayes factor for the interaction effect model was 2.6, suggesting that the observed data are 2.6 more likely to occur under the null hypothesis.

Altogether, the Bayes factor analyses supported the conclusion that clarity does not affect the magnitude of RS in any of the tested ROIs.

## Discussion

The present study aimed at investigating the impact of added sensory noise on repetition suppression. First, the results show an effect of noise level on the activity in the FFA but not in OFA or LO. This is in line with other results showing lower FFA activity (Horner and Andrews, 2009) or a weaker electrophysiological signal in electrodes over the temporal cortex (Banko et al., 2011) in response to noisy faces. Regarding the lateral occipital regions, the same studies showed even an enhanced processing in these, earlier stages of visual processing (Banko et al., 2011; Hermann et al., 2015) when exposed to noisy stimuli. However, there is also evidence for the opposite result, namely a reduced activation with increasing noise level (Malach et al., 1995) or no noise effects at all (Jemel et al., 2003; Wild and Busey, 2004). In the current study, noise had no effect on the LO, which is at odds with prior studies (Malach et al., 1995; Banko et al., 2011; Hermann et al., 2015). The chosen noise level could be one factor leading to this result. We applied 45%, whereas prior studies applied slightly higher (55%) noise levels (Hermann et al., 2015). It is possible that more noise is necessary to affect the activity of the lower level visual areas, whereas higher level visual areas are more sensitive to added visual noise. Also, in studies finding an elevated BOLD signal for noise in the LO, participants had to perform more demanding tasks like gender categorization (Wild and Busey, 2004; Banko et al., 2011), and the higher task-difficulty might led to enhanced neural activity. In contrast, the target detection task in the current study was comparatively easy and this could lead to the similar activity for noisy and clear stimuli. The fact, however, that the behavioral results show an effect of noise on participants’ performance argues against this interpretation.

Repetition suppression was found in all the tested regions, even if not always in both hemispheres (OFA and LO), regardless of the noise level. Therefore, this study joins the large body of evidence for this robust effect (for a review, see Grill-Spector et al., 2006). Interestingly, the noise added to the stimuli did not affect the magnitude of RS in any of the tested cortical areas. This suggests that the neural mechanisms driving RS are similar for clear and noisy stimuli. The exact nature of these mechanisms is highly debated currently in the literature. Specifically, while electrophysiological single-cell recording studies suggest that RS is explained by local or bottom-up mechanisms, such as fatigue (Carandini and Ferster, 1997), several current neuroimaging studies support the role of top-down mechanisms, such as predictions, in explaining RS (for a review, see Grotheer and Kovács, 2016).

Theories of predictive coding (Rao and Ballard, 1999; Friston, 2005) assume that the human central nervous system continuously makes inferences or predictions about the surrounding sensory environment and estimates the difference of the actual incoming and predicted inputs (prediction error). Many studies have suggested so far that this prediction error is reflected in the repetition-related response reduction of neurons (RS; Summerfield et al., 2008, 2011; Todorovic and de Lange, 2012; Grotheer and Kovacs, 2014; Mayrhauser et al., 2014; Grotheer and Kovács, 2015). In addition, recent theoretical (O’Reilly et al., 2012) and clinically motivated studies of predictive coding (Adams et al., 2013; Sterzer et al., 2018) suggested that the magnitude of the prediction error, therefore of RS, should depend on the precision of the predictions, as well as of the incoming sensory data. It has been suggested that precise predictions together with more noisy sensory evidence lead to enhanced prediction errors while less precise priors with more precise incoming sensory stimuli lead to reduced prediction errors (O’Reilly et al., 2012; Adams et al., 2013; Auksztulewicz and Friston, 2016; Sterzer et al., 2018). Here we modeled the precision of the sensory data by adding noise to our stimuli and we kept the precision of the predictions (i.e., the volatility of the system, Summerfield et al., 2011) constant. We asked if the modulatory effect of stimulus precision on prediction errors is reflected in the magnitude of RS. To our surprise, the results suggest that RS is insensitive to the manipulations of the precision of incoming sensory inputs, at least if the precision is modulated by adding noise to the stimuli.

The reason for the lack of modulatory effect of noise might be due to the fact that RS is the result of the interaction of multiple neural processes. While many prior human electrophysiological and neuroimaging studies explained RS in the framework of predictive coding (for a review, see Grotheer and Kovács, 2016), other studies explained RS by simpler, bottom-up, or local mechanisms (Kaliukhovich and Vogels, 2014; Vogels, 2016; Olkkonen et al., 2017; Vinken et al., 2018). Indeed, the separation of RS from its modulation by stimulus probabilities and thereby by expectation was confirmed by many studies (Larsson and Smith, 2012; Todorovic and de Lange, 2012; Grotheer and Kovács, 2015; Feuerriegel et al., 2018). We presented our Rep and Alt trials with equal probabilities; therefore, we did not modulate probabilistic expectations. Thus, it is possible that the manipulation of sensory precision affects only the modulation of RS by top-down factors, such as probabilistic expectations, but not the magnitude of RS *per se*. This would explain why we observed similar RS for noisy and clear stimuli and at the same time requires further specifically targeted studies to test. This fact, together with the currently heavily debated neural mechanisms of RS (Vinken et al., 2018), does not allow us to conclude that the precision of incoming sensory stimulation has no effect on predictive processes at all. Nonetheless, our results clearly show that the precision of the sensory input is not crucial in determining the RS magnitude *per se*.

Also, we did not observe repetition enhancement effects for the less visible, noisy stimuli which have been reported by Turk-Browne and colleagues in their study (Turk-Browne et al., 2007). However, there are several conceptual differences between their experiment and the current one. First, they used a different stimulus set (indoor and outdoor scenes) and therefore measured the BOLD response in different areas (parahippocampal place area) as we did. Second, the task was an orthogonal orientation discrimination task in our case, while an indoor-outdoor scene discrimination in the Turk-Brown study, meaning that it directed attention to the stimulus content. Third, and above all, while we used short-lagged stimulus pairs (with 500 ms average ISI) the Turk-Brown study used much longer, 3-s-long ISI with masked presentations, and it is not clear so far if these two types of presentations provoke the same neuronal mechanism or not. Altogether, these differences make the comparisons of the two studies difficult.

Manipulating the precision of sensory data was not sufficient to affect RS magnitudes at all in our study. Provided prediction errors are reflected in RS at all, posterior beliefs may be more determined by the precision of the predicted priors than by the precision of the sensory inputs. The precision of the priors can be modeled by applying stable, highly predictable or more variable, volatile stimulus sequences. Indeed, Summerfield et al. (2011) found that the repetition probability-induced modulation of RS (measured on visual evoked potentials) was present during stable stimulation segments but disappeared almost entirely when the stimulation became volatile. The aim of the current study was to test the precision of the sensory stimuli only; therefore, we did not make an effort to modulate stability/volatility here. Also, we assumed the priors to be the same for both noisy and clear conditions and for alternating and repeated stimulus pairs. In other words, we kept the probabilities of the four trial types equal and constant across the experiment. Still, we cannot exclude entirely the possibility, that the *a priori* hypotheses of the “noisy world” are different from those of a “clear world.” In other words, introducing noise to the sensory input might have had an effect on the predictive priors as well. Therefore, the lack of a modulatory effect of sensory data precision on RS suggests that future studies should manipulate sensory data precision together with the precision of prior predictions. Including precision manipulations into probabilistic prediction paradigms (e.g., as in Summerfield et al., 2008) will provide more insight into predictive processes.

## Conclusion

The findings of this study are in agreement with previous studies showing a reducing effect of noise in the region of the fusiform gyrus (Horner and Andrews, 2009; Banko et al., 2011). The enhanced activation in more lateral occipital regions found in earlier investigations (Banko et al., 2011; Hermann et al., 2015) could not be confirmed. This suggests a different sensitivity to noise of the different regions. Significant RS was present in the FFA (bilateral), right OFA, and left LO. Evidence for a modulatory effect of precision on RS could not be proved. Therefore, future studies should focus on independently manipulating the precision of prior beliefs and sensory inputs for a better understanding of its impact on predictive processes.

## Ethics Statement

Participants were fully informed about the study and gave written consents to participate. They received course credits for participation. The experiment was conducted in accordance with the guidelines of the Declaration of Helsinki and with the approval of the ethics committee of the Faculty of Social and Behavioural Sciences of the University of Jena.

## Author Contributions

S-MR, CA, and GK designed the concept of the article. S-MR and CA ran the experiments and analyzed the data. S-MR and GK wrote the article.

### Conflict of Interest Statement

The authors declare that the research was conducted in the absence of any commercial or financial relationships that could be construed as a potential conflict of interest.
